# Prevalence of Premenstrual Dysphoric Disorder among Female Students of a Medical College in Nepal: A Descriptive Cross-sectional Study

**DOI:** 10.31729/jnma.7227

**Published:** 2022-01-31

**Authors:** Reena Kumari Jha, Mina Jha

**Affiliations:** 1 Department of Physiology, Kathmandu University School of Medical Sciences, Dhulikhel, Kavre, Nepal; 2 Department of Anatomy, Janaki Medical College, Janakpur, Nepal

**Keywords:** *female*, *premenstrual dysphoric syndrome*, *premenstrual syndrome*

## Abstract

**Introduction:**

Premenstrual dysphoric disorder is a severe form of premenstrual syndrome that impairs quality of life and carries an increased risk of suicidal attempts. Hormonal changes may underlie these symptoms. The present study was conducted to find out the prevalence of premenstrual dysphoric disorder among female students of a medical college in Nepal.

**Methods:**

This descriptive cross-sectional study was conducted among 266 healthy young females in a medical college of Nepal from 21^st^ June 2021 to 31^st^ August 2021 with approval from the Institutional Review Committee 51/2021. Convenience sampling was done. Self-rated questionnaire of premenstrual symptoms screening tool was used to evaluate premenstrual dysphoric disorder. The Premenstrual Symptoms Screening Tool reflects and 'translates' categorical Diagnostic and Statistical Manual of Mental Disorders-IV criteria into a rating scale with degrees of severity. Data were analyzed using the Statistical Package for Social Sciences version 16. Point estimate at 95% confidence interval was calculated along with frequency and proportion for the binary data.

**Results:**

Out of 266 female students, we found that the prevalence of premenstrual dysphoric disorder was 10 (3.8%) (1.50-6.10 at 95% Confidence Interval).

**Conclusions:**

The prevalence of premenstrual dysphoric disorder in our study was found to be higher when compared to other similar studies.

## INTRODUCTION

Menstruation is a natural, cyclic and physiological process,^1^ but most awomen feel emotional and physical discomfort a few days prior to its onset. When these symptoms affect daily activities, it is known as premenstrual syndrome (PMS).^2^ PMS occurs during late luteal phase and abating within a few days following the menses in > 90% of menstruating women.^3,4^ The exact cause of PMS is not known.^5,6^ Premenstrual dysphoric disorder (PMDD) is a severe form of PMS and recurs for at least two menstrual cycles,^6^ affecting 3-9% menstruating women.^7^

Previous Nepalese studies have found prevalence of PMDD as 37%^8^ and 2.1 %^9^in the medical students respectively. It should be studied as it affects student's social, academic or work performance and emotional wellbeing and carries risk of depression and suicide.^9,10^

The study aims to find out the prevalence of premenstrual dysphoric disorder among female students of a medical college in Nepal.

## METHODS

A descriptive cross-sectional study was performed in two hundred sixty-six healthy young females, aged between 17 to 30 years, from 21^st^ June 2021 to 31^st^ August 2021 , in the Department of Physiology, Kathmandu University School of Medical Sciences with approval from the Institutional Review Committee of Kathmandu University School of Medical Sciences/Dhulikhel Hospital (IRC-KUSMS 51/2021). Students who were married, had irregular cycles for past six months, had a history of intake of any hormonal medication, or a major gynecological, psychological or medical problem were excluded from the study. Convenience sampling was done.

The sample size was calculated using the formula as given below:

n = Z^2^ × (p × q) / e^2^

  = (1.96)^2^ × 0.5 × (1-0.5) / (0.05)^2^

  = 384

Where,

n= required sample size
Z= 1.96 at 95% Confidence Interval (CI)
p= prevalence of premenstrual dysphoric disorder among female students of a medical college in Nepal taken as 50% for maximum sample size
e= margin of error, 5% in this study

Total female students from MBBS, BDS, B. Sc. Nursing, BNS and BPT (N)= 620

Adjusted sample size= n/[1+{(n-1)/N]}]

  = 384 / [1 + {(384-1)/620}]

  = 238.50

Thus, the minimum number of the sample size required was calculated as 239. By adding 10% as a non-response rate, the minimum sample size was 263 and a sample size of 266 was taken.

The prevalence of premenstrual dysphoric disorders was assessed using Premenstrual Symptom Screening Tool (PSST).^7,9,11,12^ The PSST reflects and 'translates' categorical DSM-IV criteria into a rating scale with degrees of severity. PSST measures the severity of premenstrual symptoms, and the diagnosis of PMDD, and is less time consuming and more practical.

Consent was obtained after explaining the risk and benefits of the study. The questionnaires were selfadministered in a classroom/Hostel with ample seating space to ensure privacy. The questionnaire was in English and all participants were educated in English medium and were able to understand the terms used in the questionnaire. The participants were allowed to ask any doubts or to clarify any terms, etc. However, none asked for any clarification. Data collection was done in a single sitting using a self-administered questionnaire which consisted of sociodemographic profile of the students, details of their menstrual cycle and the Premenstrual Symptoms Screening Tool. The PSST consists of 19 items, 14 premenstrual symptoms, and 5 functional items, in line with DSM-IV criteria. Participants were asked to rate the extent to which they experience each symptom during the late luteal phase and stopping within a few days of bleeding and the extent to which the symptoms interfere with each functional domain. Items are rated as "not at all," "mild," "moderate," or "severe."

The items of the PSST are divided into three categories to identify PMDD: (i) "core PMS" symptoms, (ii) "other PMS" symptoms, and (iii) "functional" items. The diagnostic criteria for PMDD, using the PSST, are: (a) at least 1 of 4 "core PMS" symptoms rated severe, (b) at least 4 additional of 1 to 14 PMS symptoms rated either moderate or severe, and (c) at least 1of 5 "functional" items rated severe. The diagnostic criteria for severe PMS are similar with PMDD, but less stringent: (a) at least 1 of 4 "core PMS" symptoms rated either moderate or severe, (b) at least 4 additional PMS symptoms rated either moderate or severe, and (c) at least 1 of 5 "functional" items rated either moderate or severe. The purpose of the "severe PMS" classification was to identify females who experience "clinically significant" PMS but do not meet criteria for PMDD. The rest of the subjects were considered as no or mild PMS.

Data were analyzed using IBM Statistical Package for the Social Sciences version 16 software. The data were analyzed using descriptive statistics and have been presented as means, standard deviations, frequencies, and percentages. Point estimate at 95% confidence interval was calculated along with frequency and proportion for the binary data.

## RESULTS

Out of 266 female medical students, we found that the prevalence of PMDD was 10 (3.8%) (1.50-6.10 at 95% Confidence Interval). Two hundred sixty-six students who participated in our study were between 17 and 30 years of age. The mean ages of the participants were 21.72±1.99 years with mean BMI 21.76±3.05 kg/m^2^ (Table 1).

**Table 1:** Demographic and reproductive characteristics of the participants.

Parameters	Total participants n (%)	PMDD^*^ n (%)
	266 (100)	10 (3.8)
Age (years)	21.72±1.99	21.20±1.47
BMI (kg/m2)	21.76±3.05	20.96±2.08
**Residence**		
Urban	228 (85.7)	8 (80)
Rural	38 (14.3)	2 (20)
**Age at Menarche (years)**		
<12 years	49 (18.4)	1 (10)
12-15 years	208 (78.2)	9 (90)
>15 years	9 (3.4)	0
Duration of menstrual cycle (days)	28.75±2.05	28.10±1.79
Duration of Flow (days)	4.85±1.17	5.4±1.07
**Dysmenorrhea**		
Yes	214 (80.5)	9 (90)
No	52 (19.5)	1 (10)
**Family history of PMS**		
Present	90 (33.8)	6 (60)
Absent	176 (66.2)	4 (40)
**Physical activity**		
Limited	211 (79.3)	9 (90)
Unlimited	55 (20.7)	1(10)

* PMDD-premenstrual dysphoric disorder

The most commonly reported symptom was anger/irritability 10 (100%) followed by depressed mood, lack of concentration, feeling overwhelmed or out of control (Table 2).

**Table 2:** Frequency of each Symptom in with PMDD (n=10).

Symptoms	PMDD n (%)
Anger/irritability	10 (100)
Anxiety/tension	8 (80)
Tearful/increased sensitivity to rejection	8 (80)
Depressed mood/hopelessness	9 (90)
Decreased interest in work activities	6 (60)
Decreased interest in home activities	5 (50)
Decreased interest in social activities	7 (70)
Difficulty concentrating	9 (90)
Fatigue/lack of energy	8 (80)
Overeating/food cravings	6 (60)
Insomnia (no sleep)	3 (30)
Hypersomnia (needing more sleep)	4 (40)
Feeling overwhelmed or out of control	9 (90)
Physical symptoms	5 (50)

Among participants with premenstrual dysphoric disorder, all respondents narrated that their symptoms interfered severely with work efficiency or productivity followed by home responsibility (Table 3).

**Table 3:** Interference in Work among PMDD (n=10).

Symptoms	PMDD n (%)
Work efficiency or productivity	10 (100)
Relationships with colleagues	8 (80)
Relationships with the family	7 (70)
Social life activities	7 (70)
Home responsibilities	9 (90)

## DISCUSSION

Our study was conducted among young females of Kathmandu university school of medicine sciences, attending courses of different stream. In the present study, 3.8% fulfilled the criteria of PMDD. This finding was comparable with study conducted among college students of Bhavanagar found the prevalence of PMDD was 3.7%.^13^ Study done in the teaching hospital of Kathmandu, Nepal found 2.1% respondent had PMDD.^9^ In contrast to our study, another study conducted in Palpa, Nepal found that 39.2% students had PMDD that is very higher rate than ours.^8^ Study conducted in Japan among high school athletic students found that 1.3% participants had PMDD and 8.9% had moderate to severe PMS.^14^ However Steiners reported the prevalence of PMDD was 8.3%.^12^ Thakur, et al. demonstrated that the prevalence of PMDD screened by PSST was 5.04% and the prevalence of PMDD was 4.43% by the daily record of severity of problems form (DRSP).^7^ Currently the prevalence of PMDD ranges from 3-9% in women of reproductive age.^7,15^ The wide variation in incidence of PMDD was due to geographical and cultural variation, types of study population as well as different diagnostic criteria and methodology used to diagnose PMDD.^16-8^

In overall participants, 80.5% respondents reported dysmenorrhea out of which 90% had PMDD. This figure is consistent with study conducted by Aryal, et al. reported all participants with PMDD (100%) had dysmenorrhea.^80^ A significant relationship between dysmenorrhea and PMS/PMDD exists and that indicates dysmenorrhea provokes PMDD.^8,9^

In this study the most common symptoms reported were anger/irritability (100%) followed by depression, lack of concentration, feeling overwhelmed or out of control, all three reported by 90% of the participants. This finding was similar to previous study conducted by several authors. ^9,19,20^ Positive correlation exists between the severity of PMDD and severity of depression as well as anxiety.^15^ In contradiction to our result, the most common symptoms reported among the PMDD group was fatigue/lack of energy (100%) followed by physical symptoms, irritability/anger and decreased interest in home activities.^7^ The exact cause and pathophysiology of PMS/PMDD is not known. Investigators hypothesized that fluctuation in sex steroid, altered GABA neurotransmitter system and functional sensitivity of the GABA receptor, decreased serotonin activity might involve in the pathogenesis of PMDD. GABA and serotonin are involved in regulating mood, behavior, and cognitive functions. GABA is the main inhibitory neurotransmitter in the mammalian brain and is crucial for regulation of anxiety, alertness, and seizure.^21^ In this study we have found that impairment is present in all areas of functioning, most frequently in work efficiency or productivity (100%) followed by home responsibilities (90%).

The limitation of the present study was the use of retrospective and self reported research method that could lead to recall bias. High prevalence of PMDD among young adults warrants further large-scale study to evaluate the impact of PMDD on their academic performance, quality of life, and effective intervention for alleviating the symptoms.

## CONCLUSIONS

The prevalence of Premenstrual Dysphoric Disorder in our study was found to be higher when compared to other similar studies. PMS and PMDD is an important health problem among university students. The premenstrual symptoms interfere with their work efficacy or productivity, social life and relationship with colleagues during that particular period of menstrual cycle.
